# Supplementary material for: Using honeybees for national scale long-term eDNA biomonitoring
Source: PLoS One. 2026 May 20;21(5):e0347485. doi: 10.1371/journal.pone.0347485 (PMC13189290; doi:10.1371/journal.pone.0347485)
Supplement: S1 Table — Optional questions relating to honey yield and health of the hive sampled from for the scheme that participating beekeepers can access from their online account. (PDF) [file pone.0347485.s009.pdf]

Optional question	Response options
Beekeeper status	• Amateur<br>• Commercial
Which race of bee did you use in the selected hive?	• Buckfast<br>• Hybrid<br>• *Apis mellifera carnica*<br>• *Apis mellifera mellifera*<br>• *Apis mellifera ligustica*<br>• *Apis mellifera macedonica*<br>• Other (please specify)
What is the hive type?	• Commercial<br>• Dadant<br>• Flow hive<br>• Langstroth<br>• National Deep 14 x 12<br>• National standard<br>• Smith<br>• Warre<br>• WBC<br>• Other
Number of hives kept at location over the last 12 months?	[number]
What is the estimated yield to date of honey from this hive for this year (lbs)?	[number]
If the above estimate of yield is based on multiple hives at the same locations (rather than the single hive you collected the sample from), how many hives is this?	[number]
Will you sell honey from this hive?	• Yes<br>• No
How would you rate the strength of this hive?	• Good<br>• Average<br>• Weak
For 10 hive disorders (American foulbrood, European foulbrood, stonebrood, chalkbrood, nosema, chronic bee paralysis, deformed wing virus, cloudy wing virus, sacbrood virus, varroa) beekeepers were asked:	
• Have you had it?<br>• Did you treat for it?<br>• Have you still got it?<br>• Unsure if you had it?<br>• What treatments were used and when?	• [box ticked for "yes"]<br>• [box ticked for "yes"]<br>• [box ticked for "yes"]<br>• [box ticked for "yes"]<br>• [text]
Did you give your bees additional feeding last year?	• Yes<br>• No
If you keep multiple hives at this site, over the past 12 months how many hives have you lost, if any?	[number]

If you keep multiple hives at this site, have you had any additional notable issues over the past 12 months? If yes, please give more information (e.g. failing queens, predation by wasps, poor winter survival etc.).	[text]
