# Supplementary material for: Using honeybees for national scale long-term eDNA biomonitoring
Source: PLoS One. 2026 May 20;21(5):e0347485. doi: 10.1371/journal.pone.0347485 (PMC13189290; doi:10.1371/journal.pone.0347485)
Supplement: S2 Table — These are defined based on the sample year and the land use type. (PDF) [file pone.0347485.s010.pdf]

**Year**	**UKCEH Land Cover Map resolution**	**Crops: UKCEH Land Cover® *plus*: Crops Map data**	**UKCEH Land Cover Map source**
**2018**	LCM 2015 25m rasters	LC+ Crops 2018 vector, rasterised to 25m	https://doi.org/10.5285/bb15e200-9349-403c-bda9-b430093807c7
**2019**	LCM 2015 25m rasters	LC+ Crops 2019 vector, rasterised to 25m	https://doi.org/10.5285/bb15e200-9349-403c-bda9-b430093807c7
**2020**	LCM 2019 25m rasters	LC+ Crops 2020 vector, rasterised to 25m	GB: https://doi.org/10.5285/f15289da-6424-4a5e-bd92-48c4d9c830cc
**2021**	LCM 2020 25m rasters	LC+ Crops 2021 vector, rasterised to 25m	GB: https://doi.org/10.5285/6c22cf6e-b224-414e-aa85-900325baedbd
**2022**	LCM 2021 10m rasters	LC+ Crops 2022 vector	https://doi.org/10.5285/a22baa7c-5809-4a02-87e0-3cf87d4e223a
