# Supplementary material for: Using honeybees for national scale long-term eDNA biomonitoring
Source: PLoS One. 2026 May 20;21(5):e0347485. doi: 10.1371/journal.pone.0347485 (PMC13189290; doi:10.1371/journal.pone.0347485)
Supplement: S3 Table — Information on sample returns and data uploads for 2018–2024. Hyphens represent data that was still being generated, at the time of writing. (PDF) [file pone.0347485.s011.pdf]

Sample type	2018	2019	2020	2021	2022	2023	2024
Number of samples returned (% of pack requests)	188 (75 %)	584 (75 %)	1092 (77 %)	1177 (78 %)	1475 (79 %)	739 (72%)	534 (70 %)
Number of samples with 2 km hive landcover data (% of pack requests)	243 (97 %)	752 (96 %)	1391 (98 %)	1498 (99 %)	1843 (99 %)	-	-
Number of samples with sugar/water measurements (% of samples returned)	185 (98 %)	547 (94 %)	1007 (92%)	1149 (98 %)	1363 (92 %)	711 (96 %)	532 (99 %)
Number of samples with plant species data (% of samples returned)	183 (97 %)	576 (99 %)	775 (71 %)	1121 (95 %)	1080 (73 %)	-	-
