# Supplementary material for: Using honeybees for national scale long-term eDNA biomonitoring
Source: PLoS One. 2026 May 20;21(5):e0347485. doi: 10.1371/journal.pone.0347485 (PMC13189290; doi:10.1371/journal.pone.0347485)
Supplement: S4 Table — Nomenclature of International Territory Level (ITL) region within the UK, ordered from north to south, from 2018−24. Number in parentheses indicates percentage of returned samples for that year. (PDF) [file pone.0347485.s012.pdf]

NUTS region	2018	2019	2020	2021	2022	2023	2024
Scotland	18 (10)	36 (6)	74 (7)	98 (8)	122 (8)	78 (11)	58 (11)
North East of England	3 (2)	10 (2)	17 (2)	19 (2)	27 (2)	19 (3)	15 (3)
Northern Ireland	2 (1)	11 (2)	33 (3)	25 (2)	28 (2)	16 (2)	13 (2)
North West of England	10 (5)	19 (3)	47 (4)	73 (6)	119 (8)	58 (8)	41 (8)
Isle of Man	3 (2)	7 (1)	12 (1)	7 (1)	14 (1)	7 (1)	3 (1)
Yorkshire and the Humber	6 (3)	36 (6)	69 (6)	63 (5)	85 (6)	29 (4)	40 (7)
Ireland	(0)	(0)	(0)	1 (0)	1 (0)	1 (0)	(0)
East Midlands	8 (4)	31 (5)	60 (5)	74 (6)	83 (6)	41 (6)	40 (7)
Wales	10 (5)	35 (6)	55 (5)	72 (6)	82 (6)	82 (11)	38 (7)
West Midlands	17 (9)	33 (6)	60 (5)	85 (7)	97 (7)	42 (6)	39 (7)
East of England	27 (14)	73 (12)	171 (15)	168 (14)	194 (13)	77 (10)	55 (10)
South East of England	44 (23)	144 (25)	267 (24)	233 (20)	344 (23)	153 (21)	89 (17)
London	11 (6)	43 (7)	72 (6)	87 (7)	96 (7)	28 (4)	36 (7)
South West of England	28 (15)	106 (18)	171 (15)	168 (14)	177 (12)	102 (14)	62 (12)
Channel Islands	1 (1)	2 (0)	5 (0)	5 (0)	4 (0)	6 (1)	5 (1)
