# Supplementary material for: Using honeybees for national scale long-term eDNA biomonitoring
Source: PLoS One. 2026 May 20;21(5):e0347485. doi: 10.1371/journal.pone.0347485 (PMC13189290; doi:10.1371/journal.pone.0347485)
Supplement: S5 Table — Breakdown of crop and landcover types in 2 km surrounding sampled hives. At the time of writing, habitat data was still being generated for 2023 and 2024 samples. Values given as percentages. (PDF) [file pone.0347485.s013.pdf]

	2018	2019	2020	2021	2022
Improved Grass	52.8	52.3	52.6	54.4	55.1
Winter Wheat	16.0	17.4	14.7	17.3	17.4
Winter Barley	5.8	5.7	3.8	4.2	4.4
Oilseed Rape	5.8	5.1	3.2	2.8	3.1
Other	4.2	4.7	6.5	5.5	5.3
Spring Wheat	4.2	1.5	3.0	2.5	1.5
Spring Barley	4.1	5.9	8.2	6.2	5.9
Maize	2.9	3.4	3.2	2.8	2.8
Field Beans	2.2	2.1	2.3	2.5	1.1
Potatoes	1.3	0.9	1.2	0.9	1.1
Sugar Beet	0.9	1.0	1.2	1.0	1.1
Winter Beans	0.0	0.0	0.0	0.0	0.6
Spring Beans	0.0	0.0	0.0	0.0	0.7
Overall Arable and Horticulture	27.2	26.0	24.7	25.1	23.2
Suburban	19.1	19.3	18.4	17.7	18.7
Broadleaf Woodland	8.9	9.0	10.2	10.2	10.9
Urban	6.4	6.7	5.6	6.3	6.2
Coniferous Woodland	2.2	2.2	1.6	1.6	1.8
Freshwater	1.1	1.0	1.0	1.0	1.0
Acid Grassland	1.1	1.1	1.2	1.6	1.6
Neutral Grassland	0.5	0.3	1.5	1.1	1.4
Heather	0.3	0.6	0.8	0.8	0.9
Littoral Sediment	0.3	0.4	0.4	0.4	0.5
Heather Grassland	0.2	0.5	0.6	0.6	0.8
Calcareous Grassland	0.2	0.2	0.4	0.4	0.6
Saltmarsh	0.2	0.2	0.4	0.4	0.4
Saltwater	0.1	0.2	0.1	0.2	0.2
Supralittoral Sediment	0.1	0.1	0.2	0.1	0.2
Fen Marsh Swamp	0.1	0.0	0.1	0.2	0.3
Supralittoral Rock	0.1	0.0	0.1	0.1	0.1
Littoral Rock	0.0	0.0	0.1	0.1	0.1
Bog	0.0	0.3	0.3	0.2	0.2
