# Supplementary material for: Using honeybees for national scale long-term eDNA biomonitoring
Source: PLoS One. 2026 May 20;21(5):e0347485. doi: 10.1371/journal.pone.0347485 (PMC13189290; doi:10.1371/journal.pone.0347485)
Supplement: S1 File — Details of the implementation of the citizen scientist scheme for sampling honey. (PDF) [file pone.0347485.s014.pdf]

Supplementary material for 'Using honeybees to deliver national scale long term biomonitoring'.

Supplementary Methods

Requesting a sample pack

To ensure that personal data is retained securely, and used only for communication and sample pack distribution, beekeepers interested in providing samples to the scheme do so through a tailored registered user access web portal system. Users need to create a password-protected account associated with an email address. Creating an account subscribes the beekeeper to the scheme's newsletter, which is sent out bimonthly and provides updates on the scheme's progress such as i) when sampling packs are being sent out, ii) numbers of samples received so far in a year, iii) numbers of these samples at each stage of processing and iv) summaries of results produced.

Once a beekeeper has created an online account, they are able to request a sampling pack. To do so the beekeeper must provide the location of the honey bee hive they are intending to collect the samples from and how long the hive has been at this location. This is done though an embedded interactive map allowing them to navigate to the 10 m square in which their hive is located with the architecture of the online portal allowing them to switch between different mapping options depending on their preference (e.g. OpenStreetMap or Ordnance Survey, UK; Supplementary Figure 2). Member identification of the hive's location on the map is used by the scheme to record where each sample has been collected as well as assigned a unique eight-digit number which stays with the sample throughout processing and enables it to be matched to its accompanying metadata. The eight-digit identification number ensures that beekeeper identity is protected and adds an additional layer of scientific robusticity as associated meta-data is not linked to samples during analysis. At this point the online prompts the delivery of sampling pack and packaging for its return by post (Figure 1). It is only possible for a beekeeper to request one sampling pack at a time, up to a maximum of two sampling packs per year, and it is only possible to request a second pack when their first sample has been received by UKCEH.

Sampling honey for the scheme

Following a request though the online portal each participant is sent a sampling pack containing three sterile 30 ml sample tubes pre-labelled with the unique eight-digit code and packaging for return post.

Included are sampling instructions (Supplementary Figure 3) and links to online tutorial demonstrations by a professional beekeeper. The sampling methodology focuses on the collection of honey direct from recently laid down recently capped comb storage cells which are removed with a spatula. Honey collected from recently laid down storage cells in a comb represents foraging by the hive over the last few weeks, and so is very closely tied to the collection date. In contrast, honey harvested for human consumption would be expected to have been collected after several months of hive activity and so would be of reduced value for understanding within season trends in forage plant availability. As a methodological check that sampling has occurred directly from the comb the presence of visible wax within a sample can be recorded, serving as a good indication that sampling instructions have been followed. When the sample pack is dispatched, the status of the sample in the online system is updated to "pack sent" (Figure 1). The beekeeper receives an email update every time the status of their sample changes. Each returned sample is dated by the beekeeper to identify when collection of honey occurred.

When samples are returned the eight-digit code, date and location are recorded along with the date the sample was received. A note is made of whether wax is visible in the honey and samples where this is not the case are excluded from subsequent analysis (Figure 1). Missing details (i.e. sample data) will be requested from beekeeper directly although if not provided the sample is excluded. The samples are stored separately at room temperature. The three collection tube provided by each beekeeper are designated to: 1) a long term archive stored under ambient temperature conditions in a dark room intended for future research questions; 2) eDNA barcoding for pollen analysis to quantify foraging plants utilised by the sampled honeybee hive; 3) use as part of ongoing work on post-regulation monitoring of pesticide for the UK Department for Environment, Food and Rural Affairs (Defra) for which one sample is retained for liquid chromatography-mass spectrometry (LC/MS) and gas chromatography-mass spectrometry (GC/MS) residue analysis [1, 2].

Refractometer readings

Government legislation defines honey as containing no more than 20% moisture content. Therefore, as a test of honey quality, a small amount of honey from the collection tube designated for pollen analysis is spread onto a handheld refractometer (Supplementary Figure 5). These data are uploaded to the online system for each sample and the status of the sample is updated to "sugar results added" and are provided to the beekeepers though the portal (Figure 1). A sample does not have its sugar and water content measured if it has crystallised, as the refractometer is unable to determine accurate results from crystallised hone. Crystallised honey samples are still eligible for onwards analysis.

Fermentation and microbial growth are more likely in samples with >20% moisture content: therefore, samples in this range are still archived and included in the analysis, but recorded moisture is considered if these samples act as outliers in downstream analysis.

General Data Protection for participants of the NHMS

In accordance with UK General Data Protection Regulation (GDPR) regulations, the personal details provided by participants in creating their account and requesting a sampling pack are stored within an Indicia database hosted at UKCEH and can only be accessed by members of NHMS staff with password-protected administrative rights to the website. When metadata is downloaded from the website for data analysis the file is saved on a network drive that only members of NHMS staff have access to. Assignment of a unique eight-digit code to each sample ensures that participant personal data is not visible to those processing the samples. The NHMS newsletter is sent from a password-protected account, and all participants are automatically blind carbon copied on emails, so that their identity and email address are not shared with other participants. There is an option at the bottom of the newsletter allowing participants to unsubscribe from the mailing list and participants may contact UKCEH's Data Protection Officer at any time to request their data is removed from NHMS records.

References

1. Woodcock BA, Dos Santos Pereira G, Sleep D, Oliver A, Pywell RF. Analysis of samples from National Honey Monitoring Scheme for pesticide residues to quantify pesticide exposure risk to honeybees. DEFRA report. 2022.
2. Woodcock BA, Harrison S, Pereira G, Sleep D, Savage J, Pywell RF. Assessment of neonicotinoid residues in NHMS honey samples from 2021 & 2022 - PN0809. Report to DEFRA. 2024.
